# The Extracellular Matrix in Soft Tissue Sarcomas: Pathobiology and Cellular Signalling

**DOI:** 10.3389/fcell.2021.763640

**Published:** 2021-12-09

**Authors:** Valeriya Pankova, Khin Thway, Robin L. Jones, Paul H. Huang

**Affiliations:** ^1^ Division of Molecular Pathology, The Institute of Cancer Research, Sutton, United Kingdom; ^2^ Sarcoma Unit, The Royal Marsden NHS Foundation Trust, London, United Kingdom; ^3^ Division of Clinical Studies, The Institute of Cancer Research, Sutton, United Kingdom

**Keywords:** sarcoma, extracellular matrix, integrins, discoidin domain receptors, collagen

## Abstract

Soft tissue sarcomas are rare cancers of mesenchymal origin or differentiation comprising over 70 different histological subtypes. Due to their mesenchymal differentiation, sarcomas are thought to produce and deposit large quantities of extracellular matrix (ECM) components. Interactions between ECM ligands and their corresponding adhesion receptors such as the integrins and the discoidin domain receptors play key roles in driving many fundamental oncogenic processes including uncontrolled proliferation, cellular invasion and altered metabolism. In this review, we focus on emerging studies that describe the key ECM components commonly found in soft tissue sarcomas and discuss preclinical and clinical evidence outlining the important role that these proteins and their cognate adhesion receptors play in sarcomagenesis. We conclude by providing a perspective on the need for more comprehensive in-depth analyses of both the ECM and adhesion receptor biology in multiple histological subtypes in order to identify new drug targets and prognostic biomarkers for this group of rare diseases of unmet need.

## Introduction

Soft tissue sarcomas (STS) are a group of rare cancers accounting for 1% of adult cancers and 15% of cancers in children and adolescents (Mastrangelo et al., 2012; Howlader et al., 2020). STS arise from mesenchymal stem cells (MSCs), which during development differentiate to form connective tissues in the body. Sarcomas can arise anywhere in the body with extremities being the most common site (Brennan et al., 2014). Despite the common cell of origin, STS are heterogeneous in terms of clinical presentation and molecular alterations, with over 70 unique histological subtypes with the most common diagnoses being undifferentiated pleomorphic sarcoma, liposarcoma, leiomyosarcoma and synovial sarcoma (Coindre et al., 2001; Jo and Fletcher, 2014; Blay et al., 2019). This biological heterogeneity translates into diverse clinical outcomes. Patients diagnosed with STS have varying clinical prognoses, response to therapies and survival outcomes, even within the same histological subtype (Gutierrez et al., 2007; Jo and Fletcher, 2014).

In general, patients with STS have a dismal prognosis with 5 years survival rate of ∼50% (Francis et al., 2013). Surgery remains the mainstay of clinical management for localised sarcomas; however, despite wide surgical margins, up to 50% of patients relapse after surgery (Daigeler et al., 2014; Stiller et al., 2018). Consequently, locally advanced and metastatic disease is treated in the first line setting with anthracycline-based chemotherapy, either alone or in combination with ifosfamide (Meyer and Seetharam, 2019). The treatment in the advanced setting is usually palliative with a median overall survival of less than 2 years, and only 8% of advanced STS patients remain alive 5 years after initial diagnosis (Blay et al., 2003; Harris et al., 2015; Tap et al., 2020).

STS are therefore a group of cancers of unmet clinical need with a lack of effective treatment options and poor survival across many subtypes. The successful development of novel agents for these diseases is hampered by an incomplete understanding of STS biology. In particular, the tumour microenvironment (TME) of STS is underexplored and we have a poor understanding of how interactions between sarcoma cells and the major component of the TME, the extracellular matrix (ECM), influences the clinical course of the disease. To date, most of the knowledge on ECM-tumour cell interactions in the literature comes from studies in epithelial tumours, and the ECM composition and their cognate adhesion receptor biology in mesenchymal tumours are unknown. This review will describe emerging studies describing some of the ECM components in STS and illustrate the roles of two major ECM adhesion receptor families, namely the integrins and discoidin domain receptors in the biology of STS. We will focus on a range of different subtypes including more common subtypes such as myxofibrosarcoma and liposarcoma as well as rarer subtypes such as alveolar rhabdomyosarcoma.

## The Extracellular Matrix in Soft Tissue Sarcomas

The ECM is the acellular compartment of the TME and plays important roles in cancer progression by providing adhesion sites and cell signalling cues to different cell populations within the tumour. The ECM is a complex milieu of biomolecules which comprises both polysaccharides and proteins. The Hynes laboratory has compiled a database of all theoretical proteins that constitute the ECM and its associated factors, and termed it the “matrisome” (Naba et al., 2012). The matrisome is comprised of six major categories: collagens, proteoglycans, glycoproteins, ECM-associated proteins, regulators of the ECM and secreted factors such as cytokines and growth factors. Technological advances in transcriptomic and proteomic profiling technologies have enabled characterization of the composition of matrisome in human health and disease. For instance, prognostic signatures based on matrisome profiles in several cancer types have been developed (Langlois et al., 2014; Krasny et al., 2016; Lim et al., 2017; Naba et al., 2017; Yuzhalin et al., 2018). In a study by Pearce et al., a 22 ECM protein matrix index was derived from the transcriptomic analysis of high grade serous ovarian cancer tissue specimens and subsequently applied to transcriptomic datasets of other cancer types in The Cancer Genome Atlas (TCGA) database (Abeshouse et al., 2017; Pearce et al., 2018). In this study, a high matrix index was found to be associated with worse overall survival (OS) in the TCGA sarcoma cohort of patients, highlighting that components of the matrisome may have prognostic significance in STS and might be functionally important in sarcoma tumour progression. Very few studies on the comprehensive characterization of the ECM in STS have been performed and our current knowledge of the STS ECM composition is restricted to one mass spectrometry-based proteomics study in intramuscular myxoma and grade I myxofibrosarcoma and immunohistochemical (IHC) and immunofluorescence (IF) analyses of selected ECM components in a range of different subtypes (summarised in Table 1). These studies will be discussed in the following section.

**TABLE 1:** Summary of the studies on ECM components in STS as described herein

Study	ECM components	Number of samples	Subtype breakdown	Key findings
d’Ardenne et al. (1984)	Collagen III laminin, fibronectin	60	36 STS, 24 benign tumours	Collagen III has the most variable and irregular distribution, fibronectin is abundant in nearly all cases. Laminin was strongly expressed in ASPS, malignant and benign schwannomas, neurofibroma, leiomyoma and some LMS, but not the rest of STS
Ogawa et al. (1986)	Collagen IV	103	Tumours of peripheral nerve, muscle origin, tumours of fibrous, adipose and synovial tissue, tumours of blood vessels and of uncertain histogenesis	Strong staining in STS of peripheral nerve origin and blood vessel tumours, weak to moderate staining is seen in leiomyomas, angiomyomas and LMS. SS, fibroblastic and fibrohistiocytic tumours are negative for collagen IV. In ASPS staining is around nests of cells
Ordonez et al. (1990)	Collagen IV and laminin	39	15 biphasic, 24 monophasic SS	6/15 biphasic SS show continuous staining for laminin and collagen IV around epithelioid areas. In monophasic SS and spindle-cell component of biphasic SS the staining of laminin and collagen IV is weak and focal
Haraida et al. (1996)	Collagen IV, laminin, heparan sulphate proteoglycan and fibronectin	50	20 normal adipose tissue, 13 benign lipomatous (lipomas, hibernomas and lipoblastomas) and 17 malignant (WDLPS, myxoid, spindle cell and pleomorphic LPS) tumours	Lipomas and WDLPS show predominant expression of collagen IV staining, myxoid LPS mainly stain for laminin and pleomorphic LPS mainly express fibronectin
Lian et al. (2021)	Collagen XVIII, collagen IV	42	Human: 1 aRMS and 3 eRMS	Collagen XVIII was expressed in 69% of all murine RMS, with slight predominance in aRMS compared to eRMS, α1 chain of collagen IV was expressed in 79% of RMS and α2 chain of collagen IV was mainly expressed in 67% eRMS compared to 14% of aRMS
			Murine: 15 aRMS, 6 eRMS, 17 undifferentiated sarcomas	
Stenman and Vaheri (1981)	Fibronectin	18	4 LMS, 1 fibrosarcoma, 2 neurofibrosarcoma, 2 giant-cell tumour of bone, 1 Kaposi’s sarcoma, 1 LPS, 1 malignant hemangiopericytoma	Dense network of fibronectin in all 12 sarcomas and 6 benign soft-tissue tumours
			2 leiomyoma, 1 desmoid tumour, 2 neurilemoma, 1 cavernous hemangioma	
Fukuda and Tsuneyoshi (2000)	Fibronectin	13	13 myxoid LPS	Fibronectin staining is randomly present in immature tumour cells in 5/13 samples
Guarino and Christense (1994)	Fibronectin, collagen I, III, IV, laminin and tenascin	4	2 biphasic SS and 2 monophasic SS	Strong collagen I, III and fibronectin staining in the mesenchymal but not epithelioid areas. Strong staining for laminin, collagen IV and tenascin around epithelioid areas in biphasic SS
Benassi et al. (1998)	Fibronectin, laminin, collagen IV, vitronectin	58	20 MFH, 17 MPNST, 21 SS	Fibronectin, laminin and vitronectin are detected in MFH, MPNST and in spindle cell component of SS. Collagen IV detected in MPNST and epithelioid areas of SS
Persson et al. (1988)	Laminin	10	10 ASPS	Strong laminin staining around ‘alveoli’ of tumour cells
Willems et al. (2009)	Collagens and proteoglycans	20	10 IM and 10 grade I MFS	Decorin and α1 chain of collagen VI were significantly overexpressed at mRNA and protein levels in grade I MFS versus IM

aRMS, alveolar rhabdomyosarcoma; ASPS, alveolar soft part sarcoma; ECM, extracellular matrix; eRMS, embryonic rhabdomyosarcoma; IM, intramuscular myxoma; MFH, malignant fibrous histocytoma; MFS, myxofibrosarcoma; MPNST, malignant fibrous histocytoma; mRNA, messenger ribonucleic acid; LMS, leiomyosarcoma; LPS, liposarcoma; SS, synovial sarcoma; STS, soft tissue sarcoma; WDLPS, well differentiated liposarcoma.

### Extracellular Matrix Composition in Soft Tissue Sarcomas

#### Collagens

Collagens are major proteins in the ECM and are found in all connective tissues. All types of collagens have a similar basic triple-helical structure that arises from polypeptides with a repeating motif Gly-Pro-X, where Gly is glycine and Pro is proline, and X can be any amino acid (Sweeney et al., 2008). Many STS histological subtypes, particularly those arising from fibroblasts and myofibroblasts, present with abundant extracellular collagen matrix, which is readily observed on histopathology slides with the hematoxylin and eosin stain. However, there is little characterization of specific collagen types that are present in different histological subtypes of STS (Nielsen et al., 2003). In this section we will discuss studies that have investigated the expression of different collagen types in several soft tissue tumours which are summarized in Table 2.

**TABLE 2:** Collagen types, glycoproteins and proteoglycans identified in soft tissue tumours

ECM component	Type	Histological subtype of soft tissue tumour
Collagens	Collagen III	Leiomyoma, leiomyosarcoma
	Collagen IV	Alveolar soft part sarcoma, biphasic synovial sarcoma, hibernoma, leiomyoma, lipoma, neurofibroma, malignant schwannoma, myxoid liposarcoma, schwannoma, well-differentiated liposarcoma
	Collagen VI	Intramuscular myxoma, myxofibrosarcoma
	Collagen XII	Myxofibrosarcoma
	Collagen XIV	Myxofibrosarcoma
	Collagen XVIII	Embryonic and alveolar rhabdomyosarcoma
Glycoproteins	Fibronectin	Leiomyoma, leiomyosarcoma, neurofibroma, malignant schwannoma, schwannoma, synovial sarcoma
	Laminin	Alveolar soft part sarcoma, angiosarcoma, biphasic synovial sarcoma, hibernoma, leiomyoma, leiomyosarcoma, lipoma, neurofibroma, myxoid liposarcoma, schwannoma, well-differentiated liposarcoma
Proteoglycans	Biglycan	Myxofibrosarcoma
	Chondroitin sulphate	Intramuscular myxoma, myxofibrosarcoma
	Decorin	Myxofibrosarcoma
	Heparin sulphate	Intramuscular myxoma, myxofibrosarcoma
	Keratin sulphate	Intramuscular myxoma, myxofibrosarcoma
	Lumican	Intramuscular myxoma, myxofibrosarcoma
	Prolargin	Myxofibrosarcoma
	Proteoglycan 4	Myxofibrosarcoma

There are at least 29 types of collagen, which are classified into three classes. Fibrillar collagens represent the most abundant collagen class in the body, with collagen type I, II, III, V, XI, XXIV and XXVII belonging to this class (Bella and Hulmes, 2017). Expression of collagen type III was investigated in formalin-fixed paraffin-embedded (FFPE) specimens of 36 malignant and 24 benign soft tissue tumours by IHC (d’Ardenne et al., 1984). Collagen type III was present in the stroma of most tumours analysed, and the intensity of the staining varied between histological subtypes. In all 13 leiomyosarcoma cases, which are tumours of smooth muscle origin, there was a strong pericellular staining of collagen III throughout the tumour, which was similar to the staining found in benign leiomyomas. In contrast, in the tumours of peripheral nerve origin (6 neurofibroma, 6 schwannoma and 2 malignant schwannomas), the staining was weak and irregular. It remains to be determined if the differential collagen III staining observed is due to distinct cellular origin (smooth muscle versus nerve) of these subtypes and if collagen III has a role in the growth and progression of the different soft tissue tumours.

Another class of collagens is the network forming collagens. The unique characteristics of this class of collagen is the retention of NC-terminal domains at N- and C-termini of the collagen helix, which in fibrillar collagens are removed in post-translational processing (Knupp and Squire, 2003). The presence of these domains allows head-to-head linkage of collagen fibers and their arrangement in a sheet-like structure. Collagen IV is the main network-forming collagen and is localized exclusively to the basement membrane (BM), which is a specialized form of the ECM providing compartmentalization of tissues in the body (Jayadev and Sherwood, 2017). In soft tissue tumours, expression of collagen IV was investigated by IHC on FFPE samples in pan-soft tissue tumours and synovial sarcoma-specific studies (Ogawa et al., 1986; Ordóñez et al., 1990; Guarino, 1993; Guarino and Christensen, 1994; Haraida et al., 1996). Ogawa et al. (1986), analysed samples from 103 soft tissue tumours, where schwannomas, neurofibromas, malignant schwannomas and tumours of smooth muscle origin (leiomyomas, angiomyomas and leiomyosarcomas) were the most common histological subtypes represented. Strong collagen IV staining was observed around tumour cells in all cases of schwannomas, neurofibromas and malignant schwannomas. All three cases of leiomyomas showed weak to moderate staining and all five cases of angiomyoma had moderate staining around individual tumour cells. Eight out of ten leiomyosarcomas were weakly positive for collagen IV. In contrast, tumours of fibrous origin (desmoid-type fibromatosis, fibrosarcoma, dermatofibroma and malignant fibrous histiocytoma cases) were negative for collagen IV. In tumours of adipose tissue, only one well-differentiated liposarcoma showed cytoplasmic and pericellular staining for collagen IV, whilst four myxoid and 1 high-grade myxoid (formerly “round cell”) liposarcoma were negative. Moderate to strong staining was observed lining the tumour nests in all five cases of alveolar soft part sarcoma. In three cases of biphasic synovial sarcoma, collagen IV was found surrounding epithelioid areas, and three cases of monophasic synovial sarcoma had no collagen IV stain. Similarly, in a study by Ordóñez et al. (1990), 6 out of 15 biphasic synovial sarcomas showed collagen IV positivity around epithelioid areas, while spindle cell areas of biphasic synovial sarcomas and monophasic synovial sarcomas were negative. Thus, collagen IV production in synovial sarcoma might depend on the cellular differentiation of the tumour; however, the identity of the collagen-producing cells is not clear. In a study by Haraida et al. (1996), collagen IV positivity was analysed in normal adipose tissue, 13 benign and 17 malignant adipocytic tumours. Strong collagen IV expression was seen around normal adipose cells as well as in all benign tumours. Two well-differentiated liposarcoma cases and none of the remaining 15 malignant tumours, representing myxoid and pleomorphic liposarcoma subtypes, were positive for collagen IV. Overall, collagen IV is differentially present in soft tissue tumours, with tumours of peripheral nerve, smooth muscle origin and benign adipocytic tumours generally being abundant in collagen IV. In contrast, tumours of fibrous origin and some liposarcoma subtypes typically lack collagen IV expression.

Multiplexins are the third class of collagens which are basement membrane-associated with multiple triple-helix domains that have interruptions (Myllyharju, 2004). This class is characterised by two specific features, functional and cleavable non-collagenous domains and structural features of proteoglycans. Collagen XVIII is a multiplexin, which consists of three α1 XVIII chains and has heparan sulphate (HS) proteoglycan side chains (Halfter et al., 1998). Type XVIII collagen levels were investigated in a series of specimens from human alveolar rhabdomyosarcoma and embryonic rhabdomyosarcoma and genetically-engineered mouse models of alveolar rhabdomyosarcoma, embryonic rhabdomyosarcoma and pleomorphic rhabdomyosarcoma (Lian et al., 2021). RNA sequencing analysis was performed on 57 alveolar rhabdomyosarcomas, 57 embryonic rhabdomyosarcoma human biopsies, 4 alveolar rhabdomyosarcomas and 4 embryonic rhabdomyosarcoma human cell lines, as well as in 13 alveolar rhabdomyosarcomas and 4 embryonic rhabdomyosarcoma mouse tumours. At the mRNA level, collagen XVIII is expressed significantly higher in both human and murine alveolar rhabdomyosarcomas and embryonic rhabdomyosarcoma relative to normal muscle tissue. Additionally, expression of collagen XVIII at the protein level was observed by IHC in 1 human alveolar rhabdomyosarcoma, 3 human embryonic rhabdomyosarcomas, 14 murine alveolar rhabdomyosarcomas and 6 murine embryonic rhabdomyosarcomas. Positive staining was found in all human samples apart from 1 embryonic rhabdomyosarcoma, 86% of murine alveolar rhabdomyosarcomas and 67% of murine embryonic rhabdomyosarcoma. To determine the clinical significance of collagen XVIII expression, RNA expression data from 65 human alveolar rhabdomyosarcoma and embryonic rhabdomyosarcoma biopsies obtained from the Intergroup Rhabdomyosarcoma Study-IV was correlated with OS outcome. Kaplan-Meier survival analysis showed that the 9-year OS rate was significantly lower for patients with high collagen XVIII expression than patients with low collagen XVIII (*p* = 0.01). High collagen XVIII expression was prognostic even after adjusting for other clinical factors, however further validation in an independent cohort is required to confirm the utility of collagen XVIII gene expression as a prognostic biomarker in rhabdomyosarcoma. Overall, these findings suggest that type XVIII collagen is a potential player in rhabdomyosarcoma growth and progression, and its biological role should be further investigated.

A study by West et al. (2005), examined gene expression profiles of two type of fibroblastic soft tissue tumours comprising 13 cases of benign solitary fibrous tumours and 10 desmoid-type fibromatosis cases by DNA microarray analysis, which included analysis of the expression levels of collagen genes. Histologically, both benign solitary fibrous tumours and desmoid-type fibromatosis are composed of spindled cells embedded in collagenous matrix; however, the two tumour subtypes have different clinical behaviour (Rosado-de-Christenson et al., 2003; Hartley et al., 2004). Benign solitary fibrous tumours were originally described in the lung pleura but can occur at any soft tissue site, and 80–90% of benign solitary fibrous tumours behave in a benign manner, with up to 100% cure rate. In contrast, the other 10-20% of benign solitary fibrous tumours has a high recurrence rate and most patients with recurrent disease die within 2 years (Robinson, 2006). Desmoid-type fibromatosis is a neoplasm of intermediate (locally aggressive) biologic potential, with markedly infiltrative growth pattern (Kasper et al., 2011). Although desmoid-type fibromatosis is rarely fatal, the growth pattern of these tumours makes complete resection difficult, which leads to multiple recurrences. While both benign solitary fibrous tumours and desmoid-type fibromatosis have abundant collagenous matrix, a study by West et al., showed that benign solitary fibrous tumours and desmoid-type fibromatosis have distinct gene expression profiles of collagen and matrix-remodelling genes. Desmoid-type fibromatosis had high expression of collagen I α1 chain and collagen III α1 chain genes, which are involved in fibrotic response (West et al., 2005). Consistent with the infiltrative behaviour of desmoid-type fibromatosis, these tumours also highly expressed genes belonging to disintegrin and metalloproteinases (ADAM) and matrix metalloproteinases (MMP) families that are known to remodel the ECM and be involved in cancer cell invasion (Loffek et al., 2011). In contrast, genes of MMP family were not highly expressed in benign solitary fibrous tumours, and instead there was a high expression of collagen genes involved in BM formation and maintenance, such as collagen VI α5 chain and collagen XVII α1 chain genes (West et al., 2005). While this study is small and requires an independent validation, it shows that desmoid-type fibromatosis and benign solitary fibrous tumours have distinct expression profiles of some collagen and matrix-modifying genes which are involved in differential biological processes. However, it remains to be investigated if these collagen expression profiles may be a contributing factor in the differential clinical behaviour of desmoid-type fibromatosis and benign solitary fibrous tumours.

#### Fibronectin

Fibronectin (FN) is an ECM glycoprotein that assembles in a fibrillar insoluble matrix through a cell-mediated process where secreted FN molecules bind to a specific adhesion receptor integrin α5β1 (Singh et al., 2010). Binding to integrins allows FN molecules to interact with each other, forming thin fibrils, which cluster together into a network of thicker fibril bundles. FN is crucial for the incorporation of other ECM components, e.g., collagens, fibrillins and fibulin (Kadler et al., 2008; Sabatier et al., 2009). In addition to its role in the matrix assembly, FN mediates many biological processes such as adhesion, growth, cell migration and differentiation (Nuttelman et al., 2001; Martino et al., 2009).

The expression and distribution of FN have been analysed by IF and IHC in several histological subtypes of soft tissue tumours. A summary of the subtypes that express FN is provided in Table 2. Stenman and Vaheri (1981) visualised FN by indirect IF on fresh frozen sections from 12 malignant and 6 benign soft-tissue tumours. Strong FN staining was detected surrounding individual cells in all samples regardless of histological diagnosis and malignant status of the tumour, although FN staining intensity was slightly lower in some benign soft tissue tumours compared to the malignant samples. Another study by Fukuda and Tsuneyoshi (2000) analysed FN by indirect IF on 13 FFPE samples from myxoid liposarcoma. FN was unevenly expressed around or in the cytoplasm of immature-type lipoblasts in 5 out of 13 myxoid liposarcoma. A larger study by d’Ardenne et al. (1984), analysed FN expression in FFPE samples from 36 malignant and 24 benign soft tissue tumours by IHC. The dominant histological subtypes in the study included leiomyosarcoma, fibrosarcoma, leiomyoma, neurofibroma and schwannoma. Consistent with the Stenman and Vaheri (1981) study, FN formed a dense network in all benign tumours and in all malignant cases, apart from 1 grade III tumour of unidentified cell of origin. FN staining has also been analysed in synovial sarcoma (Guarino and Christensen, 1994; Benassi et al., 1998). Histologically, synovial sarcomas are subclassified according to the extent of cellular differentiation in a tumour. Accordingly, synovial sarcomas are classified as biphasic when a tumour consists of spindle and epithelial cells, while monophasic synovial sarcomas only comprise spindle cells (Antonescu, 2020). In a study by Guarino and Christensen (1994), FN was detected by IHC in four FFPE specimens from two biphasic and two monophasic synovial sarcomas. FN staining was seen around spindle cells in monophasic and biphasic tumours; however, epithelioid structures in biphasic tumours were negative. Similar FN staining patterns were observed in a study by Benassi et al. (1998), on 7 monophasic and 14 biphasic synovial sarcomas. While these studies are small and require validation in larger cohorts, they suggest that FN expression is not subtype specific in soft tissue tumours. Instead, FN distribution could be related to the cellular differentiation of a tumour, with strong FN expression in mesenchymal areas versus no expression in epithelioid areas. Notably, the prognostic value of FN in STS has not been assessed in the studies described above, and it should be further evaluated if intensity of FN staining correlates with a more aggressive phenotype in soft tissue tumours.

#### Laminin

Laminins are large molecular weight glycoproteins and are the major constituent of the BM. Laminins are heterotrimeric proteins that contain an α-chain, a β-chain, and a γ-chain that form a cross structure, allowing binding of other ECM molecules. At present, 5 α, 3 β, and 3 γ laminin chain genes are found in both the mouse and human genomes, and the protein products assemble to form 16 distinct laminin proteins (Miner and Yurchenco, 2004). Laminins have two primary functions, one is to interact with other ECM components and have an architectural role in the formation of the BM, and the second is to interact with cell surface receptors, namely the integrins, to relay signals from the ECM and coordinate cellular behaviors such as cell adhesion, proliferation and differentiation (Hirosaki et al., 2002).

In soft tissue tumours, expression of laminin was investigated in both pan-soft tissue tumours and subtype-specific studies (d’Ardenne et al., 1984; Persson et al., 1988; Ordóñez et al., 1990; Guarino, 1993; Haraida et al., 1996). Soft tissue tumour subtypes that have been found to express laminins are summarized in Table 2. In one study, laminin was detected by IHC in FFPE samples from 36 STS and 25 benign soft tissue tumours (d’Ardenne et al., 1984). In benign tumours, strong laminin staining was seen around tumour cells in leiomyomas, neurofibromas and schwannomas but not in fibrous histiocytoma or giant cell tumour of tendon sheath. In malignant STS, laminin was present in abundance in 6 out of 13 of leiomyosarcoma, 2 out of 2 malignant schwannoma, in the only alveolar soft part sarcoma and angiosarcoma cases included in the study. A study by Persson et al. (1988), analysed expression of several ECM proteins, including laminin, by IHC in 10 FFPE samples of alveolar soft part sarcoma. All 10 cases showed strong laminin staining around the compartmentalised nests (“alveoli”) of tumour cells. In a study by Ordóñez et al. (1990), FFPE sections from 15 biphasic and 24 monophasic synovial sarcomas were stained for laminin by IHC. In monophasic synovial sarcomas and in the spindle-cell areas of biphasic synovial sarcomas laminin staining was focal, whereas in six biphasic tumours continuous staining around epithelioid areas was observed. Haraida et al. (1996), analysed expression of laminin by IHC in FFPE samples from normal adipose tissue, 13 benign and 17 malignant adipocytic tumours. Benign lipomas and hibernomas stained positively for laminin and closely resembled the laminin staining in normal white fat cells. Malignant tumours showed heterogeneous laminin staining patterns: well-differentiated liposarcoma closely resembled the staining pattern of white fat tissue. In myxoid liposarcomas laminin staining ranged from weak to intense, while pleomorphic liposarcomas lacked laminin positivity. The similarity in laminin staining between normal adipose tissue, benign lipomas and well-differentiated liposarcoma, which generally have good clinical outcome, and heterogeneity in laminin expression patterns in myxoid liposarcomas, and lack of laminin in highly malignant pleomorphic liposarcomas might indicate that laminin has a role in the malignant transformation of adipocytes. Taken together, these studies show that laminin is an abundant ECM component in soft tissue tumours of muscle, peripheral nerve and adipose origin. However, the antibodies used in the above studies were raised against a crude mixture of laminin isoforms isolated from Engelbreth-Holm-Swarm (EHS) mouse sarcoma, and therefore it is not clear which specific isoforms of laminin are expressed in these subtypes and if they are involved in sarcomagenesis. In addition, it would be interesting to investigate whether laminin isoform expression has prognostic value in liposarcoma.

#### Proteoglycans and Glycosaminoglycans

Myxoid tumours comprise a heterogeneous group of mesenchymal neoplasms ranging from benign to malignant tumours that produce the so-called myxoid ECM (van Roggen et al., 1999). The most common benign myxoid tumour is intramuscular myxoma while aggressive angiomyxoma is a locally aggressive neoplasm. Myxoid liposarcoma, myxofibrosarcoma and extraskeletal myxoid chondrosarcoma are malignant myxoid soft tissue tumours (Baheti et al., 2015). The exact composition of myxoid ECM and whether it drives differential clinical behaviour of these tumours is not understood. Some ECM components that have been identified in myxoid ECM include collagens, proteoglycans and glycosaminoglycans (GAGs) such heparan sulphate (HS), chondroitin sulphate (CS), keratin sulphate (KS) and hyaluronic acid (HA), which is found in the majority of myxoid tumour subtypes (Willems et al., 2010). GAGs are linear polysaccharides that covalently bind core proteins to form proteoglycans, with the exception of HA that does not covalently associate with proteins.

Proteoglycans and GAGs have been analysed using Alcian blue stain as well as mass spectrometry-based proteomics in intramuscular myxoma and grade I myxofibrosarcoma, which are the most frequent histological subtypes of myxoid tumours (Willems et al., 2008; Willems et al., 2009). Table 2 provides a summary of proteoglycans and GAGs expressed in the two soft tissue tumours. Intramuscular myxoma and myxofibrosarcoma differ clinically, despite sharing many histological features. Intramuscular myxoma is a benign tumour that shows no recurrence or metastasis. In contrast, myxofibrosarcoma has a high recurrence rate and increased propensity for metastases after recurrence (Mentzel et al., 1996). Willems et al. (2008), showed that intramuscular myxoma and grade I myxofibrosarcoma contain similar amounts of HA, HS, CS and KS as determined by electrolyte controlled Alcian blue staining. In a separate study, Willems et al. (2009), performed a liquid chromatography mass spectrometry (LC-MS)-based analysis of tumour lysates from 10 cases of intramuscular myxoma and 10 cases of grade I myxofibrosarcoma. In contrast to intramuscular myxoma, grade I myxofibrosarcoma uniquely expressed collagen XII chain α1 and collagen XIV chain α1, as well as five proteoglycans (lumican, proteoglycan 4, prolargin, decorin and biglycan). Collagen chains α1, α2 and α3 of collagen VI and lumican were detected among top 30 proteins in both intramuscular myxoma and myxofibrosarcoma. Moreover, increased RNA and protein levels of collagen VI chain α1 and decorin in grade I myxofibrosarcoma relative to intramuscular myxoma were confirmed by real-time polymerase chain reaction and IHC, respectively. Taken together, these studies show that the myxoid ECM in intramuscular myxoma and grade I myxofibrosarcoma is comprised of different proteoglycan and collagen components. However, it still remains to be determined whether these differences are linked to the observed differential clinical behaviour of these tumours.

## Functional Roles of the Extracellular Matrix in Soft Tissue Sarcomas

### The Role of the Extracellular Matrix in Regulating Cell Migration in Soft Tissue Sarcomas

Although expression levels of selected ECM molecules have been analysed in several STS subtypes, the molecular interactions of ECM components and their receptors, their resulting downstream signalling events and corresponding phenotypic effects on STS cell biology are poorly understood. In a study by Benassi et al. (2009), mRNA expression of a proteoglycan neuron-glial antigen 2 (NG2), also known as chondroitin sulphate proteoglycan 4 (CSPG4) was analysed in fresh frozen primary samples of 16 malignant fibrous histiocytomas, 22 leiomyosarcomas, 11 liposarcomas and 6 fibrosarcomas. High NG2 mRNA expression was found to be an independent prognostic factor in a multivariate analysis and correlated with higher risk of postsurgical metastasis [Hazard ratio (HR) = 1.55; 95% CI, 1.08–2.23; *p* = 0.017). In a follow-up study this finding was validated in a larger cohort of 109 STS patients (HR = 1.41; 95% CI, 1.03–1.94; *p* = 0.03), suggesting that high NG2 expression might be useful in predicting metastasis development in STS patients (62). NG2 is a transmembrane proteoglycan, shown to interact with collagen VI and is involved in cell adhesion, migration and invasion in glioma (Burg et al., 1996; Yadavilli et al., 2016). Additionally, NG2 is known to be associated with functional properties of cancer stem cells in melanoma, glioblastoma, basal breast carcinoma, triple-negative breast cancer (Beard et al., 2014; Wang X. et al., 2010a; Wang X. et al., 2010b). In a study by Cattaruzza et al. (2013), the NG2–collagen VI interaction was shown to play a role in cell adhesion of leiomyosarcoma (SK-UT-1) and fibrosarcoma (HT1080) cell line models. Moreover, genetic silencing of NG2 expression with RNA interference (RNAi) significantly reduced the ability of several STS cell lines to migrate through collagen I and Matrigel gels, only when the gels were supplemented with collagen VI, suggesting that the NG2–collagen VI interaction is important for sarcoma cell invasion. To investigate downstream signaling pathways activated upon NG2–collagen VI interaction, antibody arrays were used to identify phosphorylated proteins that were present in immunosorted HT1080 NG2^+^ and NG2^−^ cells treated with collagen VI. Through this approach it was found that phosphorylation of 84 sites in 76 proteins was higher in NG2^+^ versus NG2^−^ cells interacting with Col VI, while 75 sites in 58 components were found to display reduced phosphorylated, indicating that NG2-collagen VI interaction activates multiple signal transduction cascades including cell survival– and migration–promoting pathways in this fibrosarcoma model. Drugs screens revealed the phosphoinositide 3-kinase (PI3K) pathway as a candidate for further investigation. Enhanced phosphorylation of the p85 regulatory subunit of PI3K in NG2^+^ HT1080 versus NG2^−^ HT1080 cells was observed by western blot analysis confirming activation of PI3K signaling upon NG2–collagen VI interaction. Inhibition with PI3K inhibitors, LY294002 and wortmannin, significantly reduced migration of NG2^+^ HT1080 control cells whilst migration rate of NG2^−^ HT1080 cells was not affected. Overall, this study suggests that the interaction of NG2^+^ sarcoma cells with stroma rich in collagen VI might be driving STS cell migration which may explain the observed association of NG2 expression levels with a higher risk of postsurgical metastasis. Given that the NG2–collagen VI interaction was shown to activate pro-survival and pro-migratory pathways in the HT1080 fibrosarcoma model as revealed by antibody arrays, these pathways could serve as potential targets for future therapeutic intervention for this STS subtype.

### The Role of the Extracellular Matrix in Modulating Drug Response in Soft Tissue Sarcomas

The influence of the ECM on drug response in carcinomas are documented in lung, breast and prostate cancers, and interactions with adhesion receptors can increase resistance as well as induce sensitivity to certain drugs (Sethi et al., 1999; Edmondson et al., 2016; Hanker et al., 2017). In sarcoma, this area of research is underexplored. Chemotherapy is standard of care in metastatic STS, with anthracyclines preferentially used as first-line systemic therapy. There is no standard of care for second-line chemotherapy and several options can be considered. Trabectedin is an approved second-line treatment for advanced STS and in addition to its cytotoxic mode of action, it exerts its therapeutic effect via modulation of the tumour microenvironment (Zewail-Foote and Hurley, 1999; Carter and Keam, 2010). A recent study investigated the response of commonly used first- and second-line chemotherapy drugs, including trabectedin, in patient-derived cell cultures grown on plastic and embedded in collagen-based scaffolds (de Vita et al., 2021). Patient-derived cultures were obtained by a single cell dissociation of fresh surgical specimens from one leiomyosarcoma, three dedifferentiated liposarcoma, three well-differentiated liposarcoma, three undifferentiated pleomorphic sarcoma patients. Collagen-based scaffolds were prepared by freeze-drying of bovine type I collagen which was chemically crosslinked with 1,4-butanediol diglycidyl ether (BDDGE) enabling control of scaffold porosity. Cell viability was measured after the drug exposure and apart from the 1 dedifferentiated liposarcoma case, all patient-derived cultures exhibited increased sensitivity to trabectedin when grown in collagen-based scaffolds compared to plastic. This suggests that interaction of adhesion receptors on STS cells with collagen I within the scaffold induces signalling that sensitises sarcoma cells to trabectedin. This study provides evidence that ECM-cell signalling axis might be important in determining drug response in STS, although the detailed mechanisms of action are unknown.

## Integrins in Soft Tissue Sarcomas

Cells sense and respond to the ECM via adhesion proteins such as the integrin family of receptors. Integrins participate in signalling pathways that control survival, proliferation, differentiation, shape, polarity, and motility of cells and their signalling is commonly dysregulated in cancer (Guo and Giancotti, 2004). Integrins function as heterodimers of α and β subunits (Hynes, 1987; Hynes, 2002) and in mammals, there are 24 known integrin pairs formed by a combination of 18 α and 8 β subunits. The terminal domains of extracellular regions of α and β subunits assemble to form a “head” that acts as a ligand binding site. Integrins recognize many ECM ligands and each heterodimer has a distinct ligand-binding specificity. Broadly, integrin heterodimers can be divided in subfamilies, based on ligand specificity or cell distribution, into receptors recognizing arginine-glycine-aspartate (RGD) sequences, collagen and laminin specific receptors, and those restricted to leukocytes (Hynes, 2002; Humphries et al., 2006).

Integrins exist in several conformation states with different affinities for their extracellular ligands (Mould, 1996). A bent (closed) conformation represents an inactive state, with low affinity for the ECM ligand, whereas in the extended (open) conformation, integrins are active, engage ECM ligands with increased affinity and can propagate intracellular signaling (Frelinger et al., 1988; Takagi et al., 2002). Integrins are unique receptors that signal bidirectionally. During “inside-out” signaling, association of intracellular proteins talin and kindlins with the cytoplasmic tail of the β integrin subunit induces a conformational change, leading to the separation of α and β cytoplasmic integrin domains, which positions the integrin heterodimer in a fully extended (open) conformation (Liu et al., 2000; Vinogradova et al., 2002). In the extended state, integrins have increased affinity for ECM ligands and this process is referred to as integrin activation. Inside-out signaling regulates the strength of cellular adhesion and allows integrins to transmit mechanical forces required for cell migration, ECM assembly and remodeling (Zhang et al., 1996; Leiss et al., 2008; Puklin-Faucher and Sheetz, 2009).

Integrins also signal through classical ligand-dependent or the so called “outside-in” signaling to mediate cellular responses to adhesion. The integrin heterodimers lack intrinsic enzymatic activity and rely on protein-protein interactions with intracellular components such as kinases for signal transduction. Association of integrins with its ECM ligands leads to the formation of nascent adhesions that connect ECM molecules via talin directly to the cytoskeleton (Klapholz and Brown, 2017). As adhesions mature, additional scaffolding and signaling proteins such as kindlins, focal adhesion kinase (FAK), proto-oncogene c-Src (Src) and paxillin amongst others, are recruited to form multiprotein complexes that transmit signals into the cell. Many of the cytoplasmic proteins that participate in ECM-integrin signaling have been identified and have collectively been termed the integrin adhesome (Zaidel-Bar et al., 2007; Horton et al., 2016). However, this is an *in silico* definition and, therefore, theoretical and context dependent. Determination of the actual cell-specific adhesion partners can be achieved through isolation and proteomic characterisation of integrin adhesion complexes (IACs) (Geiger and Zaidel-Bar, 2012; Horton et al., 2015). In this section, we summarise the limited number of studies focused on integrin biology in different STS subtypes.

### Pan-Soft Tissue Sarcomas Studies

Although there are only a few available studies in sarcomas, the available evidence suggests that the expression of different integrin subunits is specific to certain histological subtypes and cellular differentiation states (Barth et al., 1995; Benassi et al., 1998; Hajitou et al., 2008). A study by Barth et al. (1995), investigated the distribution of integrin subunits by IHC on fresh frozen samples from 32 cancers which included 7 rhabdomyosarcomas, 8 primitive peripheral neuroectodermal tumours and 7 Ewing sarcomas. All studied subtypes were negative for expression of integrin subunits β3, β4 and α2, and positive for β1 integrin subunit. Rhabdomyosarcoma tumours were mostly α1 and α3 negative and showed heterogenous expression of α5 and α6 subunits while Ewing sarcomas and primitive peripheral neuroectodermal tumours shared a similar integrin subunit profile and were only positive for α5 and β1 subunits. The shared integrin expression profiles suggests that Ewing sarcomas and primitive peripheral neuroectodermal tumours are likely to engage similar set of ECM components and activate comparable intracellular signalling networks. This observation is in keeping with these two subtypes now being known to represent the same entity. In contrast, the differential expression of α1, α3, α5 and α6 integrin subunits in rhabdomyosarcoma relative to primitive peripheral neuroectodermal tumours and Ewing sarcoma may be indicative of distinct integrin adhesion complexes and biological function in rhabdomyosarcoma. However, it should be noted that this study had a small sample size and the findings need to be validated in independent patient cohorts.

Benassi et al. (1998), studied the expression of α2, α5, α6, αv and β3 integrin subunits by IHC on FFPE samples from the following high-grade STS subtypes: malignant fibrous histiocytoma, malignant peripheral nerve sheath tumour and synovial sarcoma, and differential expression of integrins was observed in the distinct histological subtypes. Strong β3 integrin expression was observed in malignant fibrous histiocytoma and malignant peripheral nerve sheath tumours compared to weak expression in synovial sarcoma. In contrast, expression of integrin α2 was absent in malignant fibrous histiocytoma and minimal in malignant peripheral nerve sheath tumour. In synovial sarcoma, the patterns of integrin expression depended on the cellular differentiation of the tumour, with epithelioid areas staining stronger for integrin α2 compared to the spindle cell compartment while integrin α5 was preferentially found in spindle areas and not epithelioid.

In epithelial tumour types, elevated expression of selected integrin subunits correlates with decreased survival and metastasis development (Friedrichs et al., 1995; Adachi et al., 2000; Hosotani et al., 2002; Bates et al., 2005; Gruber et al., 2005; Hazelbag et al., 2007; McCabe et al., 2007). To evaluate whether expression of integrin α6 subunit is associated with incidence of metastasis in high-grade STS, Benassi et al. (1998), split the malignant fibrous histiocytoma, malignant peripheral nerve sheath tumour and synovial sarcoma patients based on a cut-off of 35% of integrin α6-positive tumour cells on IHC stained slides, corresponding to the median value of across all samples examined. Kaplan-Meier survival analysis showed that the 2-year disease-free survival (DFS) rate was significantly lower for patients with ≥35% integrin α6-positive cells versus patients with <35% α6-positive cells (77 vs 13%; chi-square = 22.2; *p* < 0.001). However, this finding needs to be further validated in an independent cohort. No significant correlation was found between the expression of other integrin subunits and DFS. The observed correlation of high expression of integrin subunit α6 with DFS in malignant fibrous histiocytoma, malignant peripheral nerve sheath tumour and synovial sarcoma patients is consistent with findings in studies in lung and breast cancers (Friedrichs et al., 1995; Shen et al., 2019). If validated independently, integrin α6 subunit expression might be a potentially useful prognostic biomarker of metastasis in malignant fibrous histiocytoma, malignant peripheral nerve sheath tumour and synovial sarcoma patients.

Due to the rarity and heterogeneous nature of STS, there are few studies on integrins in these diseases, and most lack independent validation. Nevertheless, efforts to characterize integrin expression patterns by IHC have been undertaken in rhabdomyosarcoma, malignant fibrous histiocytoma, malignant peripheral nerve sheath tumour and synovial sarcoma patients. Current evidence shows that patterns of integrin subunit expression is distinct between histological subtypes and cellular differentiation as in the case of synovial sarcoma, which is suggestive of differences in integrin biology in these subtypes. The following sections will discuss emerging evidence on the biology of particular integrin subunits in myxofibrosarcoma, rhabdomyosarcoma and liposarcoma.

### Myxofibrosarcoma

Myxofibrosarcoma is one of the most common STS of the elderly, and is typically found in the extremities. Myxofibrosarcomas consist of spindle cells surrounded by abundant myxoid stroma. Treatment involves wide local excision; however, it is challenging to obtain negative surgical margins due to the unusual tail-like growth pattern of these tumours, with markedly infiltrative growth along vascular and fascial planes (Waters et al., 2007; Kikuta et al., 2015; Nakamura et al., 2017). This difficulty in achieving clear margins leads to one of the highest local post-surgical recurrence rates in STS, with 50–60% patients developing local recurrences (Merck et al., 1983; Sambri et al., 2016; Kikuta et al., 2017). The metastatic rate is low (20–30%) and chemotherapy is a palliative option in metastatic disease.

While myxofibrosarcoma is characterized by abundant ECM, the significance of the interactions between myxofibrosarcoma cancer cells and the ECM is poorly understood (van Roggen et al., 1999). To investigate the molecular basis of myxofibrosarcoma, 64 primary untreated high-grade myxofibrosarcoma samples were analysed by gene expression microarrays (Okada et al., 2016). Unsupervised clustering of microarray data identified two distinct clusters with different disease-specific survival (DSS). Among the differentially expressed genes between the two clusters, the integrin α10 subunit was most strongly associated with DSS (HR = 2.46; *p* < 0.01) and distant recurrence-free survival (HR = 3.75; *p* = 0.001). The integrin α10 subunit associates with β1 integrin subunit to form a α10β1 collagen receptor, which preferentially binds collagen IV and VI (Camper et al., 1998; Tulla et al., 2001). Moreover, integrin α10 subunit mRNA levels were shown to be higher in metastatic samples compared to the primary tumour samples, suggesting integrin α10 is functionally involved in myxofibrosarcoma tumour progression (Hazelbag et al., 2007).

To assess if integrin α10 subunit is important in myxofibrosarcoma biology, the authors silenced integrin α10 expression through RNAi in patient-derived myxofibrosarcoma cell lines MFX8000 and MXF8500 which led to suppressed growth and increased apoptosis. To investigate the signalling pathways downstream of integrin α10 subunit that drive growth in myxofibrosarcoma cells, the authors showed that the nucleotide exchange factor, triple functional domain protein (TRIO), and the subunit of the mTORC2 complex, rapamycin-insensitive companion of mammalian target of rapamycin (RICTOR) are direct binding partners of integrin α10 subunit. This integrin subunit mediated its tumorigenic effects though the ras-related C3 botulinum toxin substrate 1 (RAC1)/RAC1 activated kinase 1 (PAK) and protein kinase B (AKT)/the mechanistic target of rapamycin (mTORC) pathways, with TRIO controlling cell growth through the GTPase RAC1 leading to the activation of PAK1 kinase and RICTOR mediating the activation of AKT.

To further examine if integrin α10 subunit/TRIO/RICTOR axis is important in myxofibrosarcoma *in vivo*, the authors evaluated pharmacological inhibitors of the pathway in xenograft models. A direct inhibitor of integrin α10 subunit is not available and instead they employed EHop-016 which is an inhibitor of RAC1 activation and INK128 which is a mTORC inhibitor. Either inhibitor alone or in combination suppressed tumour formation and lung metastasis in myxofibrosarcoma xenografts in immunodeficient mice compared to vehicle control*.* This study provides evidence that integrin α10 subunit may control growth and metastasis development in myxofibrosarcoma, and targeting integrin α10/TRIO/RICTOR pathway with EHop-016 and INK128 inhibitors could be clinically beneficial for myxofibrosarcoma patients.

### Rhabdomyosarcoma

Rhabdomyosarcoma is the most common STS in children less than 15 years old and it represents 50% of STS in this age group (Gurney et al., 1999). The cell of origin of this subtype is unknown but rhabdomyosarcoma cells usually show variable skeletal muscle lineage differentiation and/or are positive for skeletal muscle differentiation proteins like myogenic differentiation 1 (MyoD1) or myogenin (Hosoi et al., 1992; Sebire, 2003). The two most common subtypes of rhabdomyosarcoma are embryonic rhabdomyosarcoma and alveolar rhabdomyosarcoma, and these neoplasms have different prognoses, present at different anatomical sites and have distinct molecular features (Coffin, 1997). Embryonic rhabdomyosarcoma, which represents 80% of all rhabdomyosarcoma cases, arises in children below 10 years of age, is found in the head and neck and generally has a good prognosis. Alveolar rhabdomyosarcoma is found in the extremities and is considered a rare aggressive subtype driven by PAX3-FOXO1 and PAX7-FOXO1 translocations which are present in 80–85% of alveolar rhabdomyosarcoma patients (Barr et al., 2002). Over 70% of low-risk localized rhabdomyosarcoma cases can be cured with multimodal therapy that involves surgical resection, radiotherapy and systemic chemotherapy (Crist et al., 2001; Stevens et al., 2005; Hawkins et al., 2013). However, recurrences of primary rhabdomyosarcoma and metastasis are common, mainly in alveolar rhabdomyosarcoma, and the OS of these patients is very poor, with 3 years OS being 25–30% despite intensive systemic therapy (Breneman et al., 2003; Oberlin et al., 2008; Malempati and Hawkins, 2012; Dantonello et al., 2013). There is little advancement in developing novel targeted therapies for this group of pediatric patients, primarily due to poor understanding of molecular factors driving metastasis and recurrence in rhabdomyosarcoma.

The acquisition of motility and invasive phenotypes in cancer cells are closely related to the changes in the expression profile of adhesion molecules such as integrins. Active Notch signaling was shown to control rhabdomyosarcoma cell migration and invasion which is associated with changes in the expression of adhesion molecules, including the integrin α9 subunit (Roma et al., 2011). The inhibition of Notch signaling with the specific Notch inhibitor GSI modulated the expression of adhesion molecules in rhabdomyosarcoma cells, and importantly it downregulated integrin α9 subunit expression. Integrin α9 subunit partners with β1 integrin subunit to form α9β1 integrin receptor which has a broad ligand specificity and engages with fibronectin, tenascin-c, members of a ADAM family and other ECM ligands (Høye et al., 2012). The role of this integrin subunit in invasion was further analysed in three rhabdomyosarcoma cell lines (RH30, CW9019 and HTB82 cells). Firstly, it was confirmed that integrin α9 subunit levels are regulated by the activation of the Notch pathways and not via signaling through other pathways. Notch signaling was genetically manipulated by transfecting rhabdomyosarcoma cells with Notch ligand Delta-like protein 1 (Delta 1) to constitutively activate the pathway which increased integrin α9 subunit levels at both the mRNA and protein levels compared to the mock transfected cells. Conversely, transfection of cells with the dominant negative form of the transcriptional coactivator of Notch receptors, mastermind-like protein 1 (MAML1), reduced integrin α9 subunit expression. Delta1 overexpressing rhabdomyosarcoma cells were more invasive than the mock transfected cells and when Delta1 overexpressing cells were treated with an integrin α9 blocking antibody, the invasiveness of these cells were greatly impaired compared to the controls (Masià et al., 2012). This study provides evidence for the pro-invasive role of the integrin α9 subunit in rhabdomyosarcoma and shows that cooperation of Notch signaling with integrin α9 is an underlying mechanism of rhabdomyosarcoma cell invasion. Consequently, inhibition of the Notch pathway or blocking of integrin α9 subunit may be explored as novel therapeutic options for metastatic rhabdomyosarcoma.

### Liposarcoma

Liposarcomas are tumours of adipocytic origin and are one of the most common STS subtypes, accounting for 20% of all STS diagnoses (Dei Tos, 2000). Liposarcoma is a heterogeneous disease both histologically and in terms of clinical behaviour and is subdivided into: well-differentiated liposarcoma/dedifferentiated liposarcoma, myxoid liposarcoma and pleomorphic liposarcoma (Lee et al., 2018). Well-differentiated liposarcoma and dedifferentiated liposarcoma represent different ends of the disease spectrum. Well-differentiated liposarcoma resemble mature adipose tissue, and are typically slow growing tumours which tend to recur locally. These tumours lack metastatic potential, and have good outcome when complete excision is achieved (Singer et al., 2003; Thway, 2019). In contrast, dedifferentiated liposarcoma is an aggressive disease arising *de novo* in 90% of cases with the remaining 10% occurring due to dedifferentiation of well-differentiated liposarcoma tumours. Clinically, dedifferentiated liposarcoma is characterized by high local and metastatic recurrence rates and have a much poorer prognosis than well-differentiated liposarcoma (Henricks et al., 1997). Both well-differentiated liposarcoma and dedifferentiated liposarcoma have poor response to radiotherapy and chemotherapy, and thus it is essential to identify molecular factors driving recurrence and metastasis for the development of future targeted therapies.

Wang L. et al. (2011), proposed that the high risk of relapse in liposarcoma is due to the presence of a subpopulation of cells with cancer stem cell (CSC) properties such as self-renewal potential and ability to initiate and sustain tumour growth. In the search for such a cell population, the authors established a human liposarcoma sub-line SW872-S by repeated inoculation of the liposarcoma SW872 cell line in nude mice. SW872-S had higher tumour formation potential than the parental SW872 cell line and displayed differential adhesion properties. Fluorescence-activated cell sorting (FACS) showed that SW872-S cells had an elevated expression of the integrin α6 subunit compared to the parental cell line. High expression of integrin α6 subunit in SW872-S cells was linked to increased proliferation and enhanced colony formation in soft agar. Genetic silencing of integrin α6 subunit with RNAi significantly reduced SW872-S growth *in vitro*. Treating SW872-S xenografts with an integrin α6 subunit monoclonal antibody reduced tumour formation by 60% compared with a control antibody. To determine if increased integrin α6 subunit expression is clinically relevant, specimens from 30 liposarcoma patients (comprising dedifferentiated, pleomorphic and myxoid round cell liposarcoma diagnoses who recurred within 2 years), six samples of well-differentiated liposarcoma and normal fat tissue were analyzed for integrin α6 subunit expression by IHC. Ninety percent of recurrent cases were positive for integrin α6 subunit whereas no expression was detected in well-differentiated liposarcoma patients and normal fat tissue. These results provide evidence that integrin α6 subunit may be an important driver of tumour progression in human liposarcoma and is associated with risk of recurrence in patients.

In contrast to the abundant published literature on integrin biology in epithelial cancers, this area of research remains largely unexplored in mesenchymal tumours. The studies described above highlight the involvement of integrin α10 subunit in myxofibrosarcoma growth and lung metastasis development, the contribution of integrin α9 subunit to rhabdomyosarcoma cell invasion and the role of integrin α6 subunit in liposarcoma. Additionally, elucidation of the intracellular pathways downstream of integrins that control these aspects of sarcomagenesis may offer new drug targets for the treatment of myxofibrosarcoma, rhabromyosarcoma and liposarcoma.

## Discoidin Domain Receptors in Soft Tissue Sarcomas

In addition to the integrins, the discoidin domain receptors (DDRs) are another class of adhesion receptors that are key regulators of cell-matrix interactions. DDRs belong to the receptor tyrosine kinases (RTKs) superfamily but rather than being activated by growth factors, its ligands are collagens (Fu et al., 2013). The DDR family consists of the DDR1 and DDR2 receptors: DDR2 is represented by a single protein, whereas alternative splicing can generate five distinct DDR1 isoforms. DDR2, DDR1a, DDR1b, and DDR1c receptors encode the full length receptor which is composed of an N-terminal extracellular discoidin homology domain, a single transmembrane region, a juxtamembrane domain and a C-terminal catalytic tyrosine kinase domain. DDR1d and DDR1e encode truncated and kinase inactive receptors, respectively (Alves et al., 2001). The extracellular discoidin homology domain contains a collagen-binding region and is responsible for mediating DDR specificity for fibrillar and non-fibrillar collagens. Both DDR1 and DDR2 are activated by different types of collagen, and have some shared specificity, e.g., both are activated by collagen I–III and V (Shrivastava et al., 1997; Vogel et al., 1997). Additionally, DDRs have differential ligand preferences, e.g., only DDR1 binds collagen IV, while DDR2 but not DDR1 is activated by collagen X (Shrivastava et al., 1997; Vogel et al., 1997; Leitinger, 2003; Leitinger and Kwan, 2006). In contrast to ligand-dependent dimerization of prototypical RTKs, DDRs exist as constitutive dimers (Noordeen et al., 2006; Mihai et al., 2009). Collagen binding to DDRs induces clustering of dimerised DDRs receptors and autophosphorylation of tyrosine residues in the cytoplasmic kinase domain (Iwai et al., 2013; Iwai et al., 2014). DDRs have a unique activation mechanism, where in contrast to the rapid activation and transient signalling of the classical RTKs, DDR activation by collagen is slow and when the receptor is fully active, it maintains sustained signalling for days (Shrivastava et al., 1997; Vogel et al., 1997).

There is evidence that DDR1 is preferentially expressed in normal and malignant epithelial cells, while DDR2 is highly expressed in some cells and tissues of mesenchymal origin (Johnson et al., 1993; Alves et al., 1995; Alves et al., 2001; Labrador et al., 2001; Olaso et al., 2001; Sakamoto et al., 2001; Stuart et al., 2003; Goldsmith et al., 2004; Heinzelmann-Schwarz et al., 2004; Morales et al., 2005). A few studies show that DDRs are expressed in cancers of mesenchymal origin. In phosphoproteomic screens of STS cell lines and tissue, it has been shown that the embryonic rhabdomyosarcoma cell line RD18 and malignant rhabdoid tumour cell line A204 express phosphorylated DDR1 and DDR2 but neither of receptors were expressed in the leiomyosarcoma cell line SK-LMS-1 (Bai et al., 2012). DDR1 has also been shown to be expressed in four synovial sarcoma cell lines SYO-1, HS-SY-II, YaFuSS and 1273/99 but silencing of DDR1 expression with RNAi did not alter cell proliferation (Qiao et al., 2017). Most of our current knowledge of the biology of DDRs in STS is based on the HT1080 fibrosarcoma cell line model (Wasinski et al., 2020).

### Role of Discoidin Domain Receptors in Fibrosarcoma

Fibrosarcoma represents a constellation of different malignant neoplasms of fibroblasts (e.g., infantile fibrosarcoma, low-grade fibromyxoid sarcoma) or is otherwise a non-specific morphologic pattern of sarcoma (e.g., fibrosarcomatous change in dermatofibrosarcoma protuberans). These highly aggressive tumours originate from transformed fibroblasts in collagen-rich areas such as tendons and fascias of the deep soft tissue or bone periosteum (Jo and Fletcher, 2014; Kalil, 2015). Histologically, fibrosarcoma is composed of spindle-shaped fibroblasts typically arranged in herringbone patterns. There are several types of fibrosarcoma: infantile/congenital-type fibrosarcoma is common in children under 1 year of age and is a slow growing subtype while adult-type fibrosarcoma is highly aggressive, very rare in children and has a median diagnosis age of 50 (Fletcher et al., 2002; Folpe, 2014). Molecular studies of fibrosarcoma are limited and it is currently unclear what molecular aberrations drive this disease. Fibrosarcoma is characterised by an abundant collagen matrix, which suggests that the ECM is important for the biology of this histological subtype.

To investigate the role of collagen-binding DDR1b and DDR2 receptors in fibrosarcoma, Wasinski et al. (2020), engineered human HT1080 fibrosarcoma cells that express either of these two receptors. Ectopic expression of either DDR1b or DDR2 alone did not alter tumour growth *in vitro* and *vivo*. However, receptor expression promoted enhanced tumour growth when cells were embedded within a collagen I gel and injected in mice. This result however was not reproduced in 2D *in vitro*, when DDR1b and DDR2 expressing cells were cultured on collagen I coated wells versus plastic. Taken together, these results suggest that in conjunction with binding to collagen I, structural organisation of collagen fibrils could influence DDR activation in fibrosarcoma cells. DDRs have been shown to be involved in metastasis in experimental models of lung and gastric cancers (Valencia et al., 2012; Yuge et al., 2018). Wasinski et al., evaluated this property in a fibrosarcoma experimental lung metastasis assay. Macroscopic examination of lung tissue as well as sequencing for the Alu sequence revealed HT1080 metastatic colony formation regardless of DDR2 expression. However induction of DDR1b expression significantly reduced the number of observed metastatic colonies (Wasinski et al., 2020). Thus, DDR1 appears to inhibit the ability of fibrosarcoma cells to colonise lungs in the HT1080 xenograft model.

To investigate if DDR1b and DDR2 signal through differential pathways in HT1080 xenografts, the authors checked the status of the mitogen-activated protein kinase (MAPK), AKT/PI3K and the Hippo pathways in tumours formed by DDR expressing HT1080 cells embedded within collagen I gels (COLI). No differences in phosphorylated extracellular signal-regulated kinase (ERK1/2) levels were detected in both +DDR1b/+COLI and +DDR2/+COLI tumours compared to tumours that do not express DDRs, suggesting that growth in this model is not mediated by MAPK/ERK signalling. Increased phosphorylated-AKT levels were observed only in +DDR2/+COLI tumours compared to −DDR2/+COLI, while no difference was seen for +DDR1b/+COLI tumours versus −DDR1b/+COLI tumours, supporting the role of AKT pathway in DDR2 signalling but not in DDR1b. The authors also examined the activation of the Hippo pathway by evaluating the expression of its core components: macrophage stimulating 1 (MST1), macrophage stimulating 2 (MST2) and large tumour suppressor kinase 1 (LATS1). Differential signalling was observed downstream of DDR1b and DDR2 in the HT1080 xenografts. MST2 was consistently downregulated downstream of both +DDR2/+COLI and +DDR1b/+COLI tumours compared to tumours that do not express DDRs. Overall, +DDR1b/COLI tumours showed reduced levels of LATS1 and MST1 compared to −DDR1b/COLI, while no difference was observed between +DDR2/+COLI and −DDR2/+COLI tumours, however the result was not consistent across biological replicates. Additionally, levels of kidney and brain expressed protein (KIBRA), a negative regulator of the Hippo pathway, were significantly reduced only in +DDR2/+COLI cells compared to −DDR2/+COLI and not in the context of +DDR1b/COLI versus −DDR1b/COLI (Wasinski et al., 2020). Although these data suggest that DDR1b and DDR2 differentially regulate the Hippo pathway, these alterations did not translate in clear differences in total and phosphorylated levels or the nuclear versus cytoplasmic localisation of the major effector of the Hippo pathway—Yes-associated protein (YAP), so the link between DDRs and the Hippo pathway in fibrosarcoma needs to be further investigated. Taken together, this study shows that activation of DDR1b and DDR2 in a 3D collagen matrix promotes growth of fibrosarcoma cells by activating differential pathways. Additionally, ectopic expression of DDR1b but not DDR2 suppressed experimental lung metastasis in this fibrosarcoma model. HT1080 cells carry a mutation in isocitrate dehydrogenase 1 (IDH1), whilst this aberration has not been detected in mutational analysis of fibrosarcoma patient samples (Sirvent et al., 2003; Li et al., 2015; Yang et al., 2020). Although molecular studies on fibrosarcoma are limited and more patient samples need to be analysed, this discrepancy between HT1080 cells and patient profiles is the main difficulty in using HT1080 as a faithful model to recapitulate fibrosarcoma disease. It is therefore, desirable to derive and characterise new preclinical fibrosarcoma models to confirm the role of the DDRs in this disease.

Given that DDR1b and DDR2 appear to have key roles in fibrosarcoma progression, drugs that target these RTKs may represent useful therapies. Tyrosine kinase inhibitors (TKIs), such as imatinib, nilotinib and dasatinib, are known to have a broad specificity and target multiple tyrosine kinases, including DDRs (Hantschel et al., 2008). Imatinib was originally developed against ABL kinase to treat chronic myeloid leukemia (CML), and nilotinib and dasatinib are second generation ABL kinase inhibitors used against imatinib-resistant CML. Chemical proteomic and biochemical studies have shown that these three compounds bind and inhibit DDR1 and DDR2 (Bantscheff et al., 2007; Day et al., 2008). Although the study by Wasinski et al., was performed in one fibrosarcoma cell line model and needs to be further investigated in additional patient-derived models, it is possible that TKIs such as imatinib, nilotinib and dasatinib may be considered for repurposing as DDR inhibitors for preclinical and clinical studies in the treatment of fibrosarcoma.

## Concluding Remarks and Future Directions

In recent years, there have been some efforts to map the composition of both the ECM and adhesion receptors in STS. However, much more work is required to understand the biological significance of ECM-tumour cell interactions in this group of rare diseases. One issue that remains to be addressed is the lack of a comprehensive characterization of the ECM composition in STS. The ECM is composed of hundreds of different proteins and polysaccharides, however, only the most common ECM proteins have been investigated in STS to date. Recently, proteomics methods to comprehensively profile the matrisome have been developed and are widely used to examine carcinoma-specific ECM proteomes (Naba et al., 2012; Krasny et al., 2016; Krasny et al., 2018; Socovich and Naba, 2019; Krasny et al., 2020; Milighetti et al., 2021). Moving forward, it will be important that such proteome-wide approaches are similarly applied to a broad range of STS subtypes in order to better characterise the composition and role of these molecules in tumour progression (Noujaim et al., 2016; Burns et al., 2020). Furthermore, such studies may lead to the development of new stromal-based biomarkers for prognostication or prediction of treatment outcomes in sarcomas.

Investigations of the significance of sarcoma cell interactions with the ECM requires an understanding of adhesion receptor biology in STS. This is still in its infancy but the emerging data described above indicate that both the integrins and DDRs have important functional roles in tumour progression and metastasis. Herein, we have summarised the published evidence on integrin and DDR biology in STS, but these studies are largely based on small sample sizes that require further validation in independent patient cohorts and patient-derived preclinical models. Moreover, these studies are limited to a handful of STS subtypes, and future research should expand to other histological subtypes.

In conclusion, we anticipate that future collaborative research into ECM-sarcoma cell interactions will help bridge the gap in knowledge of STS disease pathophysiology and shed light on the intracellular signalling pathways driving tumour initiation, progression and spread. This new understanding could lead to innovative avenues for novel targeted therapies and biomarkers that would inform future clinical trial development and ultimately improve clinical management for this group of diseases of unmet need.
